# Differences in emergency department visits and hospitalization between German and Dutch nursing home residents: a cross-national survey

**DOI:** 10.1007/s41999-024-00975-2

**Published:** 2024-04-29

**Authors:** Alexander M. Fassmer, Sytse U. Zuidema, Sarah I. M. Janus, Falk Hoffmann

**Affiliations:** 1 Division of Outpatient Care and Pharmacoepidemiology, Department of Health Services Research, School VI - School of Medicine and Health Sciences, Carl von Ossietzky Universität Oldenburg, Oldenburg, Lower Saxony Germany; 2 Department of Primary and Long-Term Care, University of Groningen, University Medical Center Groningen, Groningen, The Netherlands

**Keywords:** Nursing home residents, Hospitalization, Emergency department visits, Germany, The Netherlands

## Abstract

**Purpose:**

Assessing and comparing German and Dutch nursing home perspectives on residents’ hospital transfers.

**Methods:**

Cross-sectional study among German and Dutch nursing homes. Two surveys were conducted in May 2022, each among 600 randomly selected nursing homes in Germany and the Netherlands. The questionnaires were identical for both countries. The responses were compared between the German and Dutch participants.

**Results:**

We received 199 German (response: 33.2%) and 102 Dutch questionnaires (response: 17.0%). German nursing homes estimated the proportion of transfers to hospital during 1 year to be higher than in Dutch facilities (emergency department visits: 26.5% vs. 7.9%, *p* < 0.0001; hospital admissions: 29.5% vs. 10.5%, *p* < 0.0001). In German nursing homes, the proportion of transfers to hospital where the decision was made by the referring physician was lower than in the Dutch facilities (58.8% vs. 88.8%, *p* < 0.0001). More German nursing homes agreed that nursing home residents are transferred to the hospital too frequently (24.5% vs. 10.8%, *p* = 0.0069). German nursing homes were much more likely than Dutch facilities to believe that there was no alternative to transfer to a hospital when a nursing home resident had a fall (66.3% vs. 12.8%, *p* < 0.0001).

**Conclusion:**

German nursing home residents are transferred to hospital more frequently than Dutch residents. This can probably be explained by differences in the care provided in the facilities. Future studies should, therefore, look more closely at these two systems and examine the extent to which more intensive outpatient care can avoid transfers to hospital.

## Key summary points

**Aim:**

What is the difference in the frequency of transfers to hospital between German and Dutch nursing home residents?

**Findings:**

In German nursing homes, residents are transported to hospital more often than in the Netherlands (e.g., at least one emergency department visit during last year: 26.5% vs. 7.9%, *p* < 0.0001). General practitioners in Germany are less involved in the transfer decision than elder care physicians in the Netherlands.

**Message:**

The differences are probably mainly due to differences in the health and care systems (e.g., very strong gatekeeper function of the general practitioner/elder care physician in the Netherlands).

## Introduction

In Europe, there were approximately 3.4 million long-term care beds in nursing homes in 2020 [1]. Demographic changes and the aging population will require a large increase in the number of nursing home residents in almost every country [2]. Due to increased frailty and vulnerability [3, 4], nursing home residents have a higher need for medical care [5]. This may also be reflected in a higher risk of (acute) transfers to hospital [6, 7]. Many of these transfers are deemed potentially avoidable [8, 9].

Hospital transfer numbers can vary significantly between different countries [10, 11] and major differences were found not only worldwide, but even between neighboring countries. A recent systematic review examined how hospitalizations from nursing homes differ between Germany and the Netherlands [12]. Both in the first 6 months of nursing home residency and in the last 6 months of life, the proportion of nursing home residents in Germany who were hospitalized was higher than in the Netherlands (e.g., last 30 days: Germany 48.6% to 58.0% vs. Netherlands 8.0% to 15.7%).

However, these differences need to be interpreted in the context of how healthcare and primary care are organized for residents in the two neighboring countries. In Germany, long-term care is financed by long-term social care insurance. In 2021, 793,000 residents lived in German nursing homes [13, 14]. The residents receive medical care from general practitioners (GPs) who make home visits to the facilities. Further medical care can be provided by other specialists in private practice (e.g., ophthalmologists, urologists), but in most cases, they must be visited by the residents in their practices. Long-term care in the Netherlands is financed by a general income tax. In 2020, there were 115,000 residents living in Dutch nursing homes [15]. Medical care differs according to the type of nursing home. In “Verpleeghuizen” (type 1 nursing homes, majority of facilities), specially trained elder care physicians (ECPs) are employed directly by the providers. In the Netherlands, elder care medicine is a distinct discipline specializing in the long-term care of frail older people [16]. In “Verzorgingshuizen” (type 2 nursing homes, small-scale living facilities, many of which face closure following reform in 2015 [17]), GPs in private practice are responsible for providing medical care. In general, specialist treatment is not provided in the outpatient setting in the Netherlands [18].

These variations in the organization of care between German and Dutch nursing homes might result in differing care processes between both countries. However, the aforementioned systematic review provided only an indirect comparison between the two countries containing studies with different methods and study years [12]. Furthermore, it cannot explain the reasons for those large differences. In summary, more in-depth information around hospital transfers from nursing homes from Germany and the Netherlands is missing.

Therefore, the present cross-national study investigates the frequency of hospital transfers among German and Dutch nursing home residents, on the one hand, and compares underlying care processes, such as the involvement of GPs or ECP in transfer decisions, on the other hand.

## Methods

### Study design and population

This study is part of a larger public health project investigating how differences in the organization of healthcare between Germany and the Netherlands affect patient outcomes and health professionals (“Comparison of healthcare structures, processes and outcomes in the Northern German and Dutch cross-border region I (CHARE-GD I)”). The interdisciplinary project consists of four sub-projects, one of which focuses on medical care in nursing homes.

**Fig. 1:**
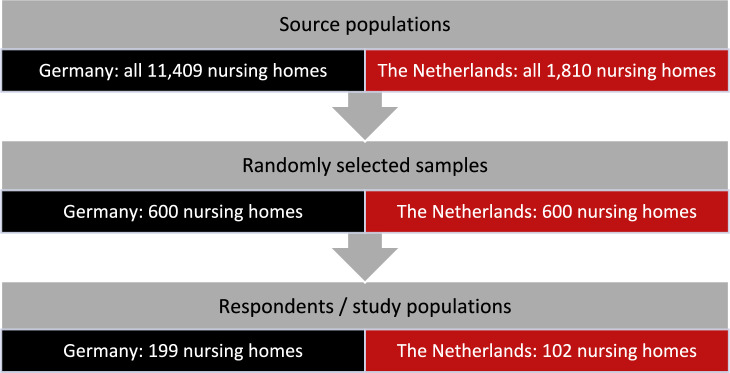
Flow chart of the study populations

Nursing homes in Germany and the Netherlands were surveyed in a cross-sectional study. As the German source population, we identified all 11,409 nursing homes listed in the Care Navigator provided by the Federal Association of Local Health Insurance Funds ("AOK Pflege-Navigator", as of January 2022). For the Netherlands, a list of all existing 1810 nursing home locations (as of February 2022) was compiled manually. We used the Caremap of the Netherlands (“Zorgkaart Nederland”), an initiative of the Dutch patient federation (patiëntenfederatie Nederland). As Zorgkaart Nederland does not differentiate between type 1 and type 2 facilities, both types were included in the list. In both Germany and the Netherlands, a simple random sample of 600 nursing homes per country was selected and surveyed by mail in May 2022 (see Fig. 1). The survey was preferably addressed to the nursing staff management/head of nursing if the name was available by a manual search. If the name of the facility administration/nursing home director or the executive board was known, this was used instead. Only if no contact person could be found, the questionnaire was addressed to the current nursing staff management/head of nursing (without personal salutation). Nursing homes could participate either in paper form (with stamped envelopes) or online via a web link or QR code (SoSci Survey). Further information on the methodological procedure can be found elsewhere [19].

Participation in this study was completely voluntary; data were collected anonymously. For both surveys, we obtained waivers from the Medical Ethics Committee of the [Carl von Ossietzky University of Oldenburg in Germany] (2022–012) and from the Medical Ethics Review Board of the [University Medical Center Groningen in the Netherlands] (2022/035).

### Content of the questionnaire

The pre-tested questionnaire included questions about medical care in nursing homes, use of emergency departments and hospitalization, and end-of-life care. For this study, we first asked participants to estimate the proportion of residents from their facility who had at least one emergency department visit and hospital admission during last year, respectively. Second, the proportion of hospital transfers (emergency department visits and hospital admissions) from the facility where the decision was made by the GP/ECP was estimated. Respondents were also asked to indicate what proportion of residents who were hospitalized did the GP/ECP see within the first week of discharge (4 options: 0–25%, 26–50%, 51–75%, 76–100%). Third, we asked whether (a) nursing home residents were too frequently transferred to the hospital, (b) there was often no alternative to hospital transfer after a nursing home resident falls, and (c) telemedicine consultations could be helpful for medical care in nursing homes. The participants had to answer these questions on a 5-point Likert scale ranging from ‘0 = totally disagree’ to ‘4 = totally agree’. Fourth, the nursing homes were asked if they used (a) a fixed emergency protocol in case of an acute deterioration in a resident's condition and (b) a fixed transfer protocol to ambulance/hospital. They also had to state if they actually used telemedicine support for the care of their residents (yes or no answer category in all three cases).

Furthermore, we asked participants for the following characteristics: their age, sex, number of care places in the nursing home, location of the facility (rural ≤20,000; semi-urban between >20,000 and ≤100,000; urban >100,000 inhabitants), distance from the nearest hospital with emergency department (in kilometers [km]), and number of years working in the current position in the nursing home (nursing staff management/head of nursing, facility administration/nursing home director, executive board, other), respectively.

### Statistical analyses

Analyses were performed using descriptive statistics. Continuous data are presented as means, standard deviation (SD), median and interquartile range (IQR). For categorical data, frequencies were calculated. For rating on the 5-point Likert scale, the proportions for the categories ‘totally disagree’ and ‘disagree’ as well as ‘totally agree’ and ‘agree’ are presented in summarized form. All assessed proportions were compared between German and Dutch participants by Mann–Whitney *U* test. Categorical variables were compared using Chi-square tests (*χ*^2^-Test). Since not all participants answered every question in the questionnaire, the analyses were restricted to subjects with no missing values given in the respective questions. Statistical analysis was performed with SAS 9.4 (SAS Institute Inc., Cary, NC, USA).

## Results

### Nursing home characteristics

199 German nursing homes responded to the questionnaire (response: 33.2%). The average age of the respondents was 48.1 years, and most were female (79.4%). The mean distance to the nearest hospital with an emergency department was 8.7 km.

From the Netherlands, we received 102 questionnaires (response: 17.0%). The Dutch participants were slightly younger than the German ones (mean age: 44.8 years) and the proportion of females was higher (85.9%). The nearest hospital with an emergency department was on average 10.4 km away. More information on participants from both countries is displayed in Table 1.

**Table 1:** Characteristics of the respondents and the nursing homes

	German nursing homes (*N* = 199)		Dutch nursing homes (*N* = 102)	
Respondent characteristics				
Age [years]	(*n* = 193)^a^		(*n* = 99)^a^	
Mean (SD)	48.1	(10.1)	44.8	(11.9)
Median (IQR)	50.0	(40.0–56.0)	48.0	(34.0–54.0)
≤49	93	(48.2%)	55	(55.6%)
50–59	66	(34.2%)	35	(35.4%)
≥60	34	(17.6%)	9	(9.1%)
Sex	(*n* = 199)^a^		(*n* = 99)^a^	
Male	41	(20.6%)	13	(13.1%)
Female	158	(79.4%)	85	(85.9%)
Diverse	–	–	1	(1.0%)
Position in the nursing home^b^	(*n* = 199)^a^		(*n* = 100)^a^	
Nursing staff management/head of nursing	140	(70.4%)	45	(45.0%)
Facility administration/nursing home director	58	(29.2%)	26	(26.0%)
Other (e.g., executive board, quality management, ward management, nurses)	19	(9.5%)	34	(34.0%)
Years in the current position in the nursing home	(*n* = 195)^a^		(*n* = 97)^a^	
Mean (SD)	9.6	(8.4)	9.0	(9.0)
Median (IQR)	7.0	(3.0–15.0)	5.0	(3.0–15.0)
Nursing home characteristics				
Location of the nursing home	(*n* = 199)^a^		(*n* = 99)^a^	
Rural (≤20,000 inhabitants)	87	(43.7%)	34	(34.3%)
Semi-urban (>20,000–≤100,000 inhabitants)	68	(34.2%)	43	(43.4%)
Urban (>100,000 inhabitants)	44	(22.1%)	22	(22.2%)
Number of beds	(*n* = 199)^a^		(*n* = 100)^a^	
Mean (SD)	83.4	(42.3)	85.0	(75.5)
Median (IQR)	80.0	(57.0–102.0)	62.0	(33.5–112.0)
Distance to the nearest hospital with emergency department [km]	(*n* = 195)^a^		(*n* = 100)^a^	
Mean (SD)	8.7	(6.9)	10.4	(8.6)
Median (IQR)	7.0	(3.0–14.0)	9.0	(4.0–15.0)

*SD* standard deviation, *IQR* interquartile range

^a^Numbers differ because of missing values, ^b^multiple answers possible**—**not applicable

### Emergency department visits and hospital admissions

When there is an acute deterioration in a resident's condition, 47.5% of German nursing homes and 64.7% of Dutch nursing homes use a fixed emergency protocol (*p* = 0.0052). On the other hand, almost all German nursing homes (98.5%) reported using a fixed transfer protocol to the ambulance service or hospital; in the Netherlands, this was 61.0% (*p* < 0.0001). The proportion of nursing homes using telemedicine support for the care of their residents was lower in Germany than in the Netherlands (15.7% vs. 26.3%, *p* = 0.0287, see Table 2).

**Table 2:** Emergency department visits and hospital admissions

	German nursing homes (*N* = 199)		Dutch nursing homes (*N* = 102)		*p* value
Does the facility use a fixed emergency protocol in case of an acute deterioration in a resident's condition?	(*n* = 198)^a^		(*n* = 99)^a^		0.0052
Yes	94	(47.5%)	64	(64.7%)	
Does the facility use a fixed transfer protocol to ambulance/hospital?	(*n* = 198)^a^		(*n* = 100)^a^		<0.0001
Yes	195	(98.5%)	61	(61.0%)	
Does the facility use telemedicine support for the care of their residents?	(*n* = 198)^a^		(*n* = 99)^a^		0.0287
Yes	31	(15.7%)	26	(26.3%)	
Proportion of residents with at least one emergency department visit during last year	(*n* = 195)^a^		(*n* = 96)^a^		<0.0001
Mean (SD)	26.5%	(19.4)	7.9%	(7.4)	
Median (IQR)	20.0%	(10.0–40.0)	5.0%	(2.0–10.0)	
Proportion of residents with at least one hospital admission during last year	(*n* = 198)^a^		(*n* = 96)^a^		<0.0001
Mean (SD)	29.5%	(19.0)	10.5%	(10.7)	
Median (IQR)	30.0%	(15.0–40.0)	7.0%	(4.0–11.0)	
Proportion of hospital transfers (emergency department visits and hospital admissions) from the facility where the decision was made by the GP/ECP	(*n* = 192)^a^		(*n* = 95)^a^		
Mean (SD)	58.8	(27.6)	88.8	(23.0)	<0.0001
Median (IQR)	60.0	(30.0–80.0)	100.0	(90.0–100.0)	
Proportion of residents discharged after hospitalization seen by GP/ECP within first week of discharge	(*n* = 199)^a^		(*n* = 101)^a^		
0–25%	37	(18.6%)	7	(6.9%)	<0.0001
26–50%	39	(19.6%)	10	(9.9%)	
51–75%	53	(26.6%)	13	(12.9%)	
76–100%	70	(35.2%)	71	(70.3%)	

*ECP* elder care physician, *GP* general practitioner, *IQR* interquartile range, *SD* standard deviation

^a^Numbers differ because of missing values

While the German participants estimated that on average 26.5% of residents had at least one emergency department visit in the past year, the Dutch indicated 7.9% in this respect (*p* < 0.0001). A similar picture was observed for hospital admissions (Germany: 29.5% vs. Netherlands: 10.5%, *p* < 0.0001). In German nursing homes, the proportion of hospital transfers (emergency department visits and hospital admissions) where the decision was made by the GP/ECP was lower than in the Dutch facilities (58.8% vs. 88.8%, *p* < 0.0001). Similarly, in Germany, the proportion of residents discharged from the hospital who were seen by their GP within the first week was lower. In the German nursing homes, 35.2% of respondents stated that in 76–100% of hospital discharges, the GP sees the affected resident within the first week; in the Netherlands this was 70.3% (*p* < 0.0001, see Table 2).

**Fig. 2:**
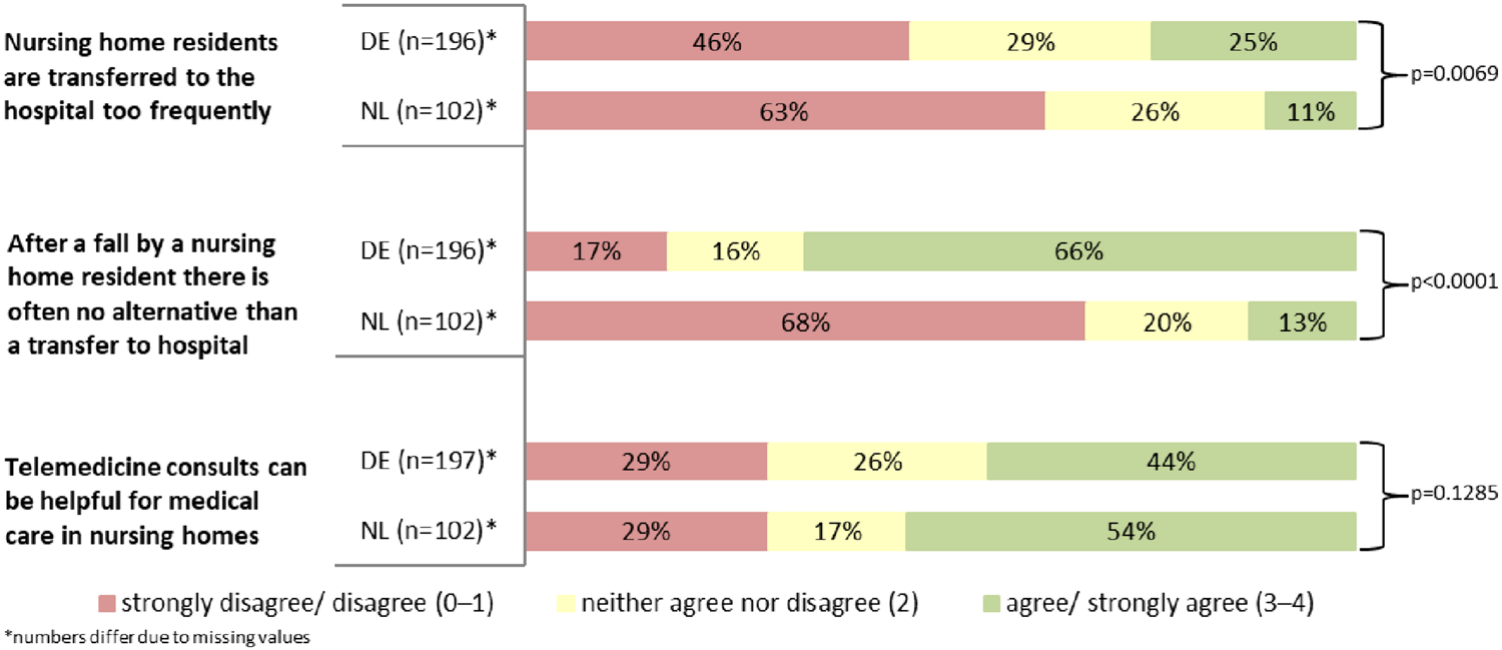
Responses to statements regarding emergency department visits and hospital admissions of nursing home residents—comparison between Germany (DE) and the Netherlands (NL)

While 24.5% of respondents in Germany agreed that nursing home residents were transferred to the hospital too frequently, the agreement in the Netherlands was 10.8%. On the other hand, the proportion of disagreement was higher in the Netherlands (62.8% vs. 46.4%, *p* = 0.0069). Two thirds (66.3%) of German nursing homes saw no alternative to a hospital transfer after a resident’s fall. In the Netherlands, only 12.8% of respondents shared this opinion (*p* < 0.0001). Nursing homes from both countries had similar views on the potential benefits of telemedicine for the medical care of nursing home residents, with slightly higher agreement in the Netherlands than in Germany (53.9% vs. 44.2%, *p* = 0.1285, see Fig. 2).

## Discussion

This cross-national study showed that German nursing home residents are transferred to hospital more often than Dutch residents. Furthermore, in Germany, the transfer decisions are less often made by the physician, and more German nursing homes feel that their residents are taken to hospital too often.

The central finding of this study, that German nursing home residents are transferred to hospital more frequently, is also consistent with the results of the aforementioned systematic review [12]. Although the proportion of German nursing home residents with hospital transfers in the last 12 months is considerably higher than in the Netherlands, it is lower than in another German study in which data were collected between 2014 and 2015 [20]. Our data collection took place 2 years after the introduction of infection control measures against the COVID-19 pandemic in nursing homes [21], which may explain the lower transfer figures for Germany. The figures we found for the Netherlands are also lower than in previous Dutch studies looking at hospitalizations of nursing home residents in the last months of life, with proportions between 15.2% [22] and 27.6% [23]. However, in both studies, the proportion of type 2 nursing homes was considerably higher than it is today in the Netherlands, which may have influenced the transfer figures. In general, hospitalizations of nursing home residents are particularly common shortly after moving to the nursing home and again at the end of life. The systematic review found higher figures for Germany compared with the Netherlands for both periods, especially at the end of residents’ lives [12]. In addition to structural differences, cultural differences may also play a role in explaining these variations between the two countries. There is more discussion about quality of life versus life-sustaining treatment in the Netherlands than in Germany and refusal of potentially distressing life-sustaining treatments is more common in Dutch nursing homes [24].

However, one of the main reasons for these differences in hospital transfers is assumed to be residents’ medical care. In Dutch type 1 nursing homes (verpleeghuizen), on the one hand, there is round-the-clock care by the directly employed ECPs and, on the other hand, all types of Dutch nursing home are regularly supported by a multiprofessional team of allied health professionals (e.g., psychologists). From the same data collection on which this study is based, we know that in German nursing homes permanently employed allied health professionals are rare [19]. This outpatient multidisciplinary care delivered in Dutch nursing homes is probably more expensive than nursing home care in Germany. However, by avoiding hospital transports [25], in the Netherlands, costs could be lower overall. Besides, we know that the GP/ECP contact frequencies are comparable with Germany [19]. Therefore, we suspect that the delivery of physician care differs in the two countries. A previous study from Germany showed that GPs are often not involved in hospital transfer decisions in nursing homes, e.g., because they cannot be reached by the facilities [26]. Meanwhile, it is known that better GP accessibility can help nursing home residents receive appropriate care earlier and thus avoid hospital transfers [8]. The present study was also able to show less frequent involvement of the physician in Germany. In the Netherlands, GPs/ECPs were involved in most hospital transfer decisions. Therefore, they may have seen other alternatives to hospitalization in case of a resident’s fall, where the majority of German nursing homes would send the resident directly to hospital.

Given that the existing literature identifies a significant proportion of hospital transfers from nursing homes as potentially avoidable [8, 27], a low number of transfers can also be seen as an indicator of good quality and well-coordinated care [28]. One indication of better coordination of care on the Dutch side could be the higher proportion of nursing homes in our study that have a fixed emergency protocol in the event of a deterioration in the condition of their residents. Although German nursing homes were more likely to have a handover protocol for the emergency services, other studies have reported that valuable information is often missing in these documents [29]. Overall, better communication, documentation, and decision support tools allow treatment without hospitalization [8]. This survey showed that Dutch nursing homes more often see a benefit in telemedical support and actually use it more often in the care of their residents. In the entire Dutch healthcare system, e-health solutions seem to play a more important role than in Germany [30, 31]. These solutions also have the potential to avoid hospitalizations of nursing home residents and reduce care costs [32].

### Limitations and strengths

Some results of this study, such as the proportions of nursing home residents with at least one hospital transfer in the past year, must be interpreted with a certain degree of caution because they are based on nursing home staff estimates. Respondents were asked to estimate data for their entire facility, but it is unclear whether participants also looked in residents’ records and how they estimated. It is possible that respondents only have current cases in mind when answering such questions, which may result in difficulties when making more general statements. Furthermore, it is possible that the nursing staff management (our preferred recipient) were not the people with the best overview in every nursing home to adequately answer our questions. However, the present study is the first to collect data on emergency department use and hospitalization of nursing home residents using the same methodology in the two neighboring countries of Germany and the Netherlands. As the primary aim of the study was to compare the frequency of hospital transfers and care processes on a nursing home level, the appropriateness of individual transfers was not assessed. Another limitation concerns the generalizability of the results. The number of cases may have been too small for some comparisons, mainly because of a lower response from the Netherlands. However, this was difficult to predict in advance. Responses from Germany and even more so from the Netherlands were not particularly high, but we previously took various measures for a higher response (including a short questionnaire, follow-up contact, among others [33]). We received responses from all German federal states as well as from all Dutch provinces. Nevertheless, a selection bias cannot be excluded in both countries. In the Netherlands, we surveyed both nursing home types (verpleeghuizen and verzorgingshuizen). While these are organized differently, we were not able to find too many differences between them in our previous article [19].

### Conclusions and implications

This study was the first to compare the frequencies of emergency department visits and hospitalization between German and Dutch nursing homes using the same methodology. German nursing home residents are more likely to be hospitalized and often GPs are not involved in transfer decisions. These results are probably mainly due to the different arrangements of general and medical care in nursing homes and the immediate availability of multiprofessional teams, which simplify the direct care of residents in the Netherlands compared with Germany. Better coordination of medical care for residents is probably the best starting point for reducing unnecessary hospital transfers. Therefore, future studies should shed more light on how many hospital transfers can be avoided through this kind of outpatient care in the nursing home and whether costs can be saved overall despite comprehensive care in the facility. In order to better understand the care processes in nursing homes, the perspectives of the professionals involved (in particular nurses and GPs/ECPs) should be compared.

## Data Availability

The dataset generated during and/or analysed during the current study are available from the corresponding author on reasonable request.
